# When Food Kills

**DOI:** 10.1371/journal.pbio.0020112

**Published:** 2004-04-13

**Authors:** John Krebs

## Abstract

Food-borne disease kills humans only rarely, although the ramifications and implications of these few deaths for science, regulators, and government are large

For the estimated 800 million people, living largely in developing countries, without enough food to eat, the main food risk is starvation. But if you ask, ‘When does food actually kill?’ in a country such as the United Kingdom, ‘Not that often’ is the short reply you would give after reading Hugh Pennington's book *When Food Kills:* BSE, E. coli, *and Disaster Science*. The two food-borne diseases that occupy much of the book, Escherichia coli 0157 and bovine spongiform encephalopathy (BSE), kill humans very rarely, although the ramifications and implications of these few deaths for science, regulators, and government are large.[Fig pbio-0020112-g001]

**Figure pbio-0020112-g001:**
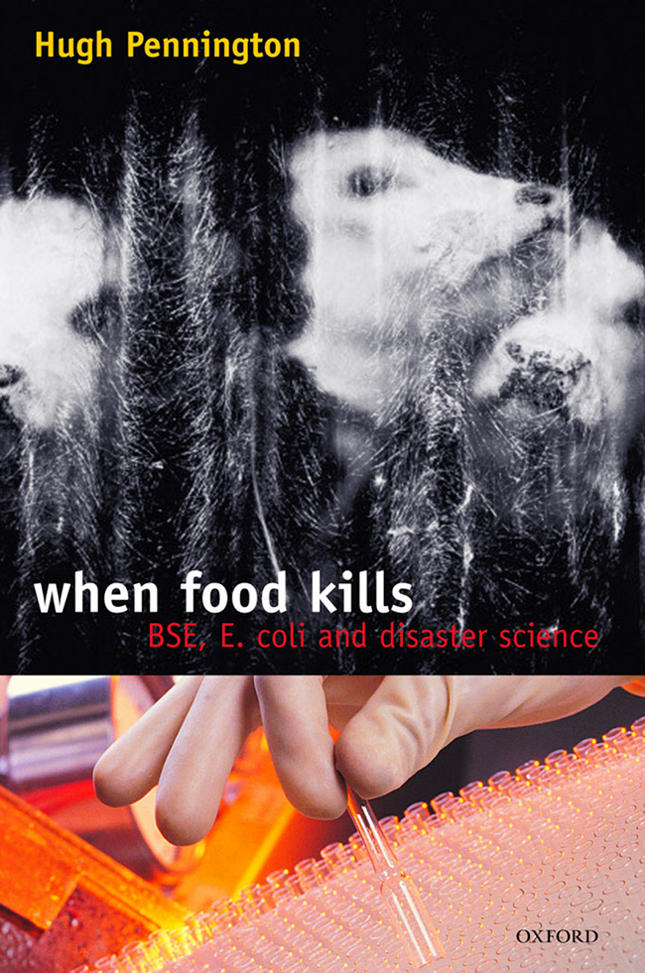


As Pennington clearly explains, there is still much uncertainty in the science of BSE, and the eventual UK death toll from the human form may be as low as a few hundred, with even the most pessimistic expert assessments putting the upper bound as fewer than 5,000. Food-borne E. coli 0157 kills fewer than a dozen people a year in the UK.

Whilst each death is a terrible tragedy and an indescribably harrowing experience for those close to the victim, these figures are small when compared with other ways in which food kills. Epidemiologists estimate that the dietary contributions to cardiovascular disease and cancer between them kill more than 100,000 people a year in Britain. Yet we hear much more about BSE and E. coli as food risks. For instance, a recent study by the King's Fund (http://www.kingsfund.org.uk/pdf/healthinthenewssummary.pdf) reports that the rate of news coverage in the UK of a death from variant Creutzfeldt-Jakob disease, the human form of BSE, is nearly 23,000 times that for a death from obesity.

In his characteristically diverting and obscurely erudite way, Pennington describes this discrepancy between public perception and magnitude of risk by referring to an article on railway accidents published in 1859 by one Dionysius Lardner. The systematic and much more revealing analyses of risk perception by psychologists such as Paul Slovic over the past 25 years do not get a mention.

In fact, one of the hallmarks of Pennington's style is his enthusiasm for taking his reader down little-known historical byways. Whether it be the drowning (possibly suicide) of King Ludwig II of Bavaria in the Starnberger See or the treatment of James Norris in Bethlehem Lunatic Asylum in 1814, Pennington has an almost endless supply of anecdotes to provide peripheral colour to his main narrative. Indeed, on some occasions his delight in the detail makes it hard to see where the main narrative is leading, although his aim is to show that similar conclusions can be drawn about risk management in food, transport, oil rigs, and other fields.

Anyone who has heard Hugh Pennington speak will know that he has a remarkably direct and engaging style, which he translates into the written word with verve. Already on page 2, he gets us into the mood by referring to a sample from a five-year-old girl sent for analysis at the start of the Lanarkshire E. coli outbreak of 1996: ‘It was a stool. The word carries the impression of firmness, even of deliberate effort in its production. Hers was not’. His laconic sense of humour is also reflected in many of the wittily irrelevant or tangential photographs. My personal favourites are ‘Her Majesty in Gloves’ on page 44 and ‘Turds on Campsite Track’ on page 101.

The Lanarkshire E. coli 0157 outbreak, which in late 1996 affected 202 people and killed eight, was very much Pennington's show. He chaired the public enquiry that led eventually to a change in the law, requiring all butchers in the UK handling cooked and raw meat to be licensed. The license itself is less important than the training in food safety management principles that precedes it. The butcher John Barr (and his staff), whose shop was the primary source of the outbreak, apparently did not know that you have to keep raw meat and ready-to-eat products separate to avoid cross-contamination with dangerous pathogens, such as E. coli 0157, that can occur in raw meat. Pennington's authoritative and blow-by-blow account shows failings not only in the butcher (who was, incidentally, Scottish Master Butcher of the Year in 1996), but also in the inspectors who had visited his shop eight times in the previous two years. They had not, apparently, picked up that Barr and his staff employed the same knives for cutting up raw and cooked meat, nor that they used a ‘biodegradable’ cleaning fluid, not realising that this is not the same as ‘biocidal’.

The second theme, BSE, is given somewhat shorter treatment. Nevertheless, Pennington goes into some detail in assessing the prion theory of transmissible spongiform encephalopathies (he argues that a nucleic acid is not also involved). He also reviews the sequence of events that led the UK government in the early 1990s to conclude that there was not likely to be a risk to human health and to be slow to change its view. This and the concluding part of the book (see below) draw heavily on the Phillips Enquiry into BSE. Although this enquiry focussed on the response of the UK government, its lessons are relevant to other countries where BSE has emerged in recent years, including many European countries, Japan, Canada, and the United States.

In his book *Mountains of the Mind*, Robert Macfarlane writes: ‘[F]or the hunter risk wasn't optional—it came with the job. I sought risk out, however. I courted, in fact paid for it. This is the great shift which has taken place in the history of risk…. [I]t became a commodity’. Pennington reflects a similar shift in attitude to food risk over the past half century or so. Back in 1938, although it was known that over 2,500 people a year in Britain died from drinking raw milk, the risk was not seen as large enough to warrant legislation to make pasteurisation compulsory. We are now used to much higher standards of food safety, and we can, as a society, enjoy the luxury of fear of relatively minor risks.

Nevertheless, there are important lessons from past failures for all involved in food safety (and in other areas of risk management), and Pennington discusses some of these in his concluding chapters. He emphasises the need to continually review the evidence underpinning risk assessments, to communicate effectively with the media, to ensure that actions to manage risks are effectively implemented and audited. Notably, he refers to the importance of inclusiveness and openness about risk and uncertainty in decision-making: ‘[I]f [this] becomes the norm, it will be possible to say that good has come out of tragedy’.

